# Pathogenesis of experimental salmonid alphavirus infection in vivo: an ultrastructural insight

**DOI:** 10.1186/s13567-015-0300-2

**Published:** 2016-01-08

**Authors:** Tharangani K. Herath, Hugh W. Ferguson, Manfred W. Weidmann, James E. Bron, Kimberly D. Thompson, Alexandra Adams, Katherine F. Muir, Randolph H. Richards

**Affiliations:** Institute of Aquaculture, School of Natural Sciences, University of Stirling, Stirling, UK; School of Veterinary Medicine, St George’s University, St. George, Grenada, West Indies; Moredun Research Institute, Pentlands Science Park, Bush Loan Penicuik, Edinburgh, UK

## Abstract

Salmonid alphavirus (SAV) is an enveloped, single-stranded, positive sense RNA virus belonging to the family *Togaviridae*. It causes economically devastating disease in cultured salmonids. The characteristic features of SAV infection include severe histopathological changes in the heart, pancreas and skeletal muscles of diseased fish. Although the presence of virus has been reported in a wider range of tissues, the mechanisms responsible for viral tissue tropism and for lesion development during the disease are not clearly described or understood. Previously, we have described membrane-dependent morphogenesis of SAV and associated apoptosis-mediated cell death in vitro. The aims of the present study were to explore ultrastructural changes associated with SAV infection in vivo. Cytolytic changes were observed in heart, but not in gill and head-kidney of virus-infected fish, although they still exhibited signs of SAV morphogenesis. Ultrastructural changes associated with virus replication were also noted in leukocytes in the head kidney of virus-infected fish. These results further describe the presence of degenerative lesions in the heart as expected, but not in the gills and in the kidney.

## Introduction

Salmonid alphavirus (SAV) is an enveloped, single-stranded, positive sense RNA (+ssRNA), virus belonging to the family *Togaviridae*. This unique group of viruses causes pancreas disease (PD) in cultured Atlantic salmon (*Salmo salar* L.) and sleeping disease (SD) in rainbow trout (*Oncorhynchus mykiss* Walbaum) [1–3]. Recently, SAV has also been detected from marine flatfish spp. in Scotland [4] and Ireland [5]. According to sequence analysis of the most variable genes; nsP3 and E2, SAVs have been classified into six subtypes (SAV 1–6) [6]. Clinical outbreaks of marine PD that cause severe economic losses to commercial aquaculture are reported from the UK (caused by SAV 1, 2, 4 and 5) [6], Ireland (caused by 1, 2, 4 and 6) [7] and Norway (caused by SAV 2 and 3) [8]. Clinical outbreaks of SD in rainbow trout in fresh water, caused by SAV 2, have been reported in many continental European countries including France, Germany, Italy, Poland and the UK [6–9].

Clinical signs of pancreas disease include lethargy, sudden in-appetence, increased presence of yellow faecal casts in the cages and fish swimming close to the water’s surface or crowding in the corners of cages. In sleeping disease, trout may also appear listless, unable to hold their position in the water column, and may lay on their side at the bottom of the cages, hence the term sleeping disease [1–3]. The sequential pathology of both PD and SD is characterised by extensive loss of pancreatic acinar tissue and inflammatory infiltration, widespread degeneration and mild to moderate mononuclear cell infiltration in atrial and ventricular myocardium and also in red and white skeletal muscles [10–12]. The mortality rate of a typical SAV outbreak can range from 5 to 60% [2], although loss associated with poor growth performance in the recovered fish and also carcass rejection at processing collectively contribute to massive loss of biomass, rendering SAV infection economically important to commercial salmon farming in Europe [13].

The replication cycle of alphavirus takes place in the cytoplasm of the host cells. The E2 envelope protein of the virus binds with host cell receptors prior to internalisation via clathrin-mediated endocytosis [14]. The low pH in the endosome triggers membrane fusion between the viral envelope and the endosome, releasing the nuclear material of virus into the cytoplasm. The alphavirus genome resembles eukaryotic mRNA, which possesses a 5′ cap and 3′ poly (A) tail and codes for early non-structural and late structural phases during replication. Initially, two-thirds of the +ssRNA genome from the 5′ end is rapidly translated to give a polyprotein, which is subsequently cleaved into non-structural protein (nSP) [15, 16]. The complementary minus strand, formed by the nSP, acts as a template for the production of genomic RNA, and also for sub-genomic RNA, which encodes the virus structural proteins. The nucleocapsid, comprising genomic RNA and nucleoprotein formed in the cytoplasm, travels towards the plasma membrane and buds out, thereby acquiring an envelope. Recently we reported observations of SAV morphogenesis in vitro, which resembled that of other alpha viruses [17].

From the six different SAV subtypes, SAV 1 and 3 are particularly associated with pathology in the cardiovascular and muscular tissue of cultured salmon [10, 11]. Although, PD has been known since the early 1980s, it has only developed into an economically significant salmonid disease since its re-emergence in 2005 [18]. Many studies have been performed to try and understand the sequential pathology and pathogenesis of the disease, but the tissue tropism of SAV is not well described. So far, sequential histopathology and virus load studies have highlighted the fact that SAV can result in different outcomes [2]. For example, the recognised target tissues, pancreas, heart and skeletal muscle, often show severe lesions, while in other tissues (e.g. gill, pseudobranch) the virus causes no observable damage, but appears to persist for long periods of time [12]. The objective of this study was to examine tissue tropism occurring in an experimental SAV 1 infection in Atlantic salmon and describe the ultrastructural changes that occur in various tissues following infection.

## Materials and methods

### Virus

Salmon alphavirus subtype-1 (SAV 1) isolate (F02-143), originally obtained from an SAV1 outbreak in 2002 in Ireland was kindly provided by Dr. David Graham, Agri-Food and Bioscience Institute, Belfast, Northern Ireland. Cultivation of the virus was performed in Chinook salmon embryo-214 (CHSE-214) cells [2, 17]. To determine the 50% tissue culture infective dose (TCID_50_) as described by Spearman and Karber [19], the stock virus was absorbed on to pre-formed CHSE-214 cells before use for the experiment (passage 9, TCID_50_/mL = 10^7.166^).

### Animals and experimental challenge

Atlantic salmon parr were obtained from Howietoun hatchery, Stirling, UK and reared at the Aquaculture research facility, University of Stirling, UK according to UK Home Office guidelines. Prior to experimental challenge, fish were screened to confirm absence of common salmon viral diseases including infectious pancreatic necrosis virus and SAV by cell culture and Real time Quantitative Reverse Transcription polymerase chain reaction (RT-qPCR).

For the experimental challenge, the fish (mean weight 27.3 ± 3.6 g (SD) were randomly divided into two groups and allocated into triplicate 20 L tanks (three tanks per group and 10 fish/tank). Fish in Group-1 were injected intraperitoneally (i.p.) with 0.1 mL of the stock SAV and fish in Group-2, were injected i.p. with CHSE-214 cell culture supernatant under benzocaine anaesthesia (40 mgL^−1^). The tanks had a freshwater flow-through system with a maximum flow rate of 500 mL min^−1^ and water temperature was maintained at 10 ± 1 °C. Fish were monitored four times daily over an 11 day experimental period to observe their behaviour and also for signs of morbidity in the tanks following UK home office animal experiment procedures.

### Sampling

At 4 dpi, and 11 dpi, three fish were sampled from each tank. Fish were euthanized by anaesthetic overdose (Benzocaine 100 mgL^−1^) and bled from the caudal vein before sampling tissues. The left-side second gill arch of each sampled fish was excised and divided into equal halves. The weight of the sample was measured and one half of the gill was suspended 1:10 w/v in Hank’s balance salt solution (HBSS) (Gibco) supplemented with 1% foetal bovine serum (FBS) and antibiotics (penicillin 100 IU mL^−1^, streptomycin 100 mg mL^−1^, kanamycin 100 mg mL^−1^) and homogenised in a Fastprep^®^ tissue homogeniser. The other half of the gill was again divided in two; one half was fixed in 2.5% glutaraldehyde in a cacodylate buffer for transmission electron microscopy (TEM) and the other half homogenised in 0.8 mL TRI-reagent^®^ (Sigma-Aldrich) for total RNA isolation. The head kidney was sampled from each fish, divided into two, weighed and processed as described above for virus isolation, using an initial dilution of 1:10 w/v, TEM and total RNA isolation. The heart was also dissected from each fish and divided along the longitudinal axis into two halves taking care to include both the atrium and the ventricle in each sample and weighed. One half was processed for virus isolation, as described for gill and kidney (using an initial dilution of 1:50 (w/v) for virus isolation) and the other half was divided into two before fixing one piece (2–3 mm^3^) for TEM and the other for RNA isolation as described earlier.

### Determination of SAV titre

The gill, heart and kidney homogenates prepared with HBSS, were centrifuged at 3500×*g* for 15 min at 4 °C. The clarified supernatants of gill and head kidney were then further diluted to prepare a 1:50 (w/v) dilution (heart tissue was already diluted 1:50 w/v). The 1:50 dilution of each tissue homogenate was titrated in triplicate using a five-fold dilution series performed in 96 well micro-titre plates. The diluent used was HBSS + 2%FBS. The plates were supplemented with freshly-prepared CHSE-214 cells and incubated for 14 days at 15 °C, 1% CO_2_ atmosphere. The development of cytopathic effect (CPE) consistent with SAV infection was observed and recorded. The titre of the virus was calculated and expressed as TCID_50_ values determined using the Spearman-Karber method [19].

### Estimation of SAV load in different tissues

Soon after collection, tissue samples in TRI-reagent (Sigma) were homogenised in a Fastprep^®^ tissue homogeniser and stored at −70 °C until extraction of RNA following the manufacturer’s instructions. The quantity of RNA obtained was measured by NanoDrop™-1000 spectrophotometer (Thermo Scientific) and the quality of RNA checked by agarose gel (1%) electrophoresis prior to reverse transcription of 1 µg of total RNA using a High Capacity cDNA synthesis kit (Applied Biosystems) according to the manufacturer’s instructions (Applied Biosystems). To estimate the SAV, RNA copy number in different tissues, RT-qPCR was performed.

The viral RNA copy number was estimated using an externally prepared SAV-specific RNA standard [20, 21]. A linearised DNA template was obtained from a conventional RT-PCR with a modified primer pair designed against the nsP1 region of the SAV genome (GenBank Accession no. AY604235.1) with a T7 RNA polymerase promoter tagged to the forward primer (5′TAATACGACTCACTATAGGGCCGGCCCTGAACCAGTT3′) and the reverse primer kept untagged (5′GTAGCCAAGTGGGAGAAAGCT3′). The PCR product was purified using a QIAquick PCR purification (Qiagen) kit and the DNA quantity was measured by Nanodrop. To obtain a large amount of in vitro transcribed “sense” RNA for downstream applications, the purified PCR product carrying T7 promoter was subjected to cycle sequencing (GATC BioTech) and then in vitro transcribed using a MEGAscript^®^ high yield transcription kit (Ambion) following the manufacturer’s instructions. The RNA transcripts obtained from in vitro transcription (cRNA) were then purified using a phenol:chloroform extraction and isopropanol precipitation. This method was chosen, based on the manufacturer’s recommendations for transcripts that encoded products less than 500 bp in size. The specificity of the amplicon was assessed by sequence analysis (GATC BioTech,) before any downstream applications. The initial number of RNA molecules per µL was estimated using Quant-iT™ RiboGreen RNA assay kit (Life Technologies) and 10 µL of cRNA containing 10^10^ copies of RNA was reverse transcribed into cDNA to prepare a standard curve. Primer efficiency (E) and the relative co-efficiency of the standard curve were optimised before use in the actual test.

The RT-qPCR to estimate virus load in the gill, head kidney and heart tissues was performed in a Mastercycler^®^ ep *realplex*^2^ (Eppendorf) qPCR machine using Absolute qPCR SYBR green Mix (Thermo Scientific) real-time chemistry. The PCR temperature profile comprised of initial activation of PCR at 95 °C for 15 min, then 40 cycles of denaturation at 95 °C for 15 s, annealing at 60 °C for 15 s, and elongation at 72 °C for 20 s. A melting curve analysis was performed to confirm the specificity of the reactions. Standard curve slopes, cycle threshold number (C_T_) vs log quantity, and PCR efficiencies (E) were calculated using the *realplex* software V2.2 (Eppendorf). The obtained absolute viral RNA copy numbers present in individual samples extrapolated in the *realplex* software were statistically validated to measure the difference between time points using a Mann-Witney U test in Minitab version 16.2.4.0.

### Transmission electron microscopy

For electron microscopy, tissues of virus-infected and control fish were collected on 4 and 11 days post-infection (dpi). These were immersed in cold 2.5% (v/v) glutaraldehyde in 100 mM sodium cacodylate buffer (pH 7.2) for 4 h at 4 °C. Samples were then rinsed in 1 M sucrose buffer for 24 h. Samples were post-fixed with 1% buffered osmium and dehydrated in a graded acetone series at 22 °C, before embedding in low-viscosity resin. Ultra-thin sections of the tissues were cut and stained with 1% aqueous uranyl acetate followed by Reynold’s lead citrate before being placed on 200-mesh Formvar-coated copper grids. The sections were examined in an FEI Tecnai Spirit G2 Bio Twin Transverse electron microscope (Olympus).

## Results

None of the fish died in either the control group or in the challenge group, during 11 day challenge period. However, fish in the challenge group appeared darker in colour than the unchallenged control fish from 3 dpi and separated themselves from the rest of the fish while swimming, whereas all of the control fish continued to swim together in the water column. Furthermore fish in the challenge tanks appeared more excitable from 3 dpi onwards, but this activity subsided by 8 dpi.

### Virus isolation and quantification

Of the three replicate fish sampled from each experimental tank on 4 and 11 dpi, only two fish were processed for virus isolation. Of the four heart, kidney and gill samples taken at each time point, two hearts, three kidneys and all gills produced a CPE on CHSE-214 cells at 4 dpi, and one heart, all kidneys and all gill produced a positive CPE at 11 dpi (Table 1). The CPE was characterised by cell rounding and sloughing off from the surface of the CHSE-214 cell monolayer. The virus titre (Log10 TCID_50_/g) in gill and kidney were high in day 11 compared to day 4 post infection, and the titre value ranged from 4.4 to 6.6 in heart, 5.0 to 7.1 in kidney, and 5.2 to 6.4 in gills. None of the samples taken from control fish produced a CPE or toxic effect on the CHSE-214 cells.

**Table 1:** **Cell culture isolation of salmonid alphavirus from different tissues of experimentally challenged Atlantic salmon parr**.

Day post challenge	Log 10 TCID_50/_gram tissue				
	Tank	Fish	Heart	Kidney	Gill
Day 4	Tank 1	Fish 1	Negative	Negative	5.2
		Fish 2	Negative	5.9	5.7
	Tank 2	Fish 4	4.4	5.0	5.4
		Fish 5	5.7	6.6	5.5
Day 11	Tank 1	Fish 1	6.6	7.1	6.4
		Fish 2	Negative	5.3	5.7
	Tank 2	Fish 4	Negative	6.1	5.3
		Fish 5	Negative	6.6	6.1

The 50% tissue culture infective dose (TCID_50_) of heart, gill and head kidney homogenate prepared from samples obtained from intraperitoneally challenged fish on 4 and 11 days post-challenge was estimated on 11 days post-inoculation on to CHSE-214 cells.

### Virus loading with RT-qPCR

**Figure 1:**
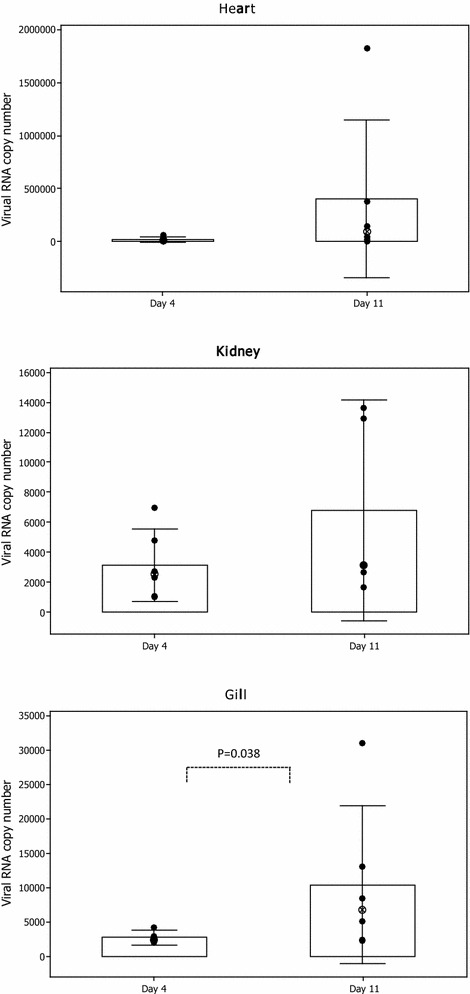
**Salmonid alphavirus replication in different tissues of experimentally challenged Atlantic salmon parr.** SAV genomic RNA replication in heart, gill and head kidney (*n* = 6) obtained on 4 and 11 days post-challenged was measured using an absolute qRT-PCR assay detecting nsP1. *p = 0.05, ⊗ Median.

All three tissues taken from the infected fish were found to be positive for viral RNA by RT-qPCR. The median copy number of viral RNA was lower at 4 dpi than 11 dpi in all three tissues examined. Moreover, the viral RNA copy number in heart and gill appeared significantly different between the two time points (Figure 1).

### Electron microscopy

**Figure 2:**
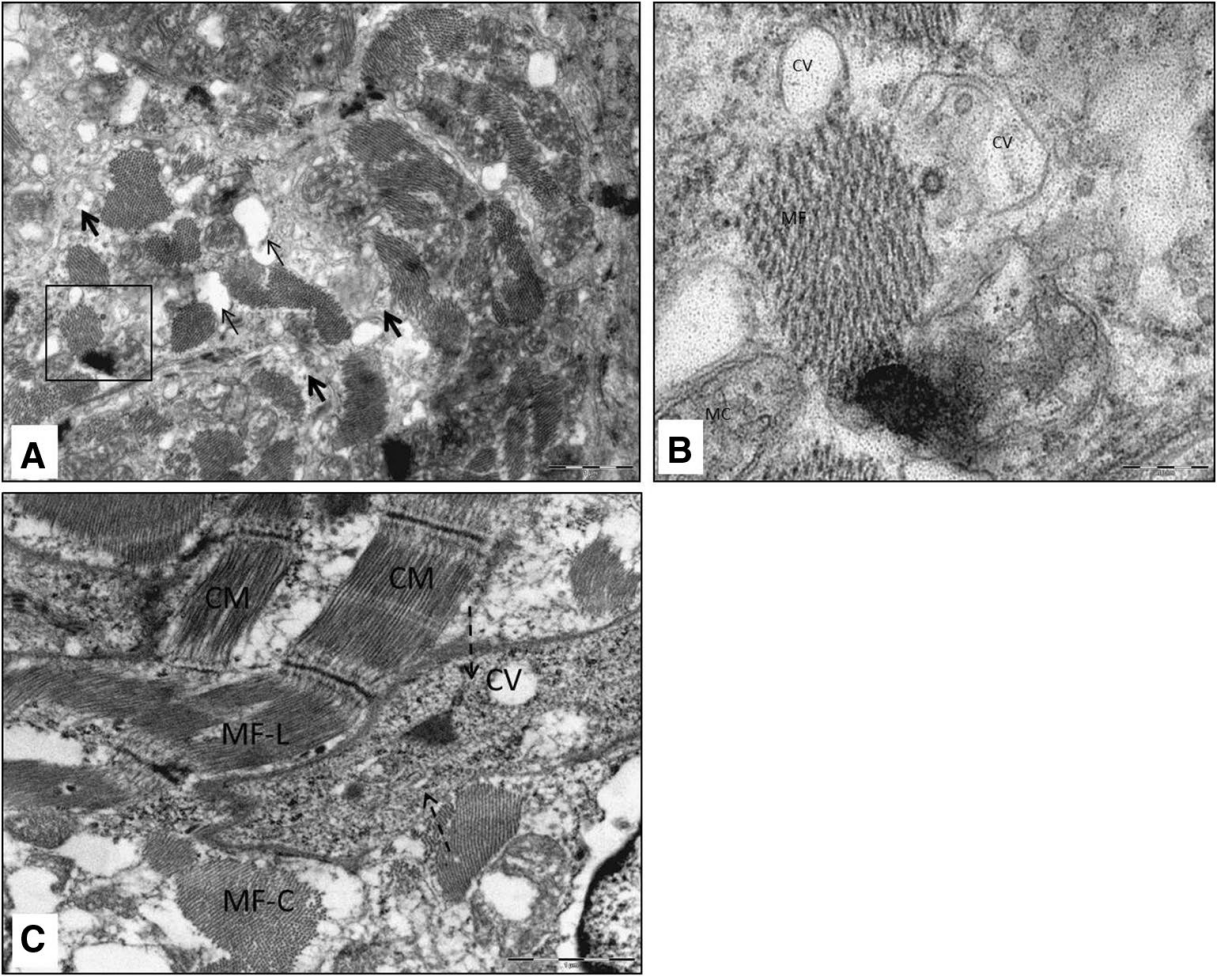
**Transmission electron micrograph of SAV-infected Atlantic salmon myocardium 11** **days post-infection.** **A** Cross section of cardiomyocyte with damaged myofilaments (thick arrow) and numerous cytoplasmic vacuoles (thin arrow); **B** higher magnification of the blocked square in (**A**) showing intact myofibrils (MF), cytoplasmic vacuoles (CV) and damaged mitochondria (MC); **C** intact cardiomyocyte with myofibrils, longitudinal section (MF-L) and cross section (MF-C) and damage of cardiomyocyte (dashed arrow) with cytopathic vacuoles (CV) and no myofibrils.

**Figure 3:**
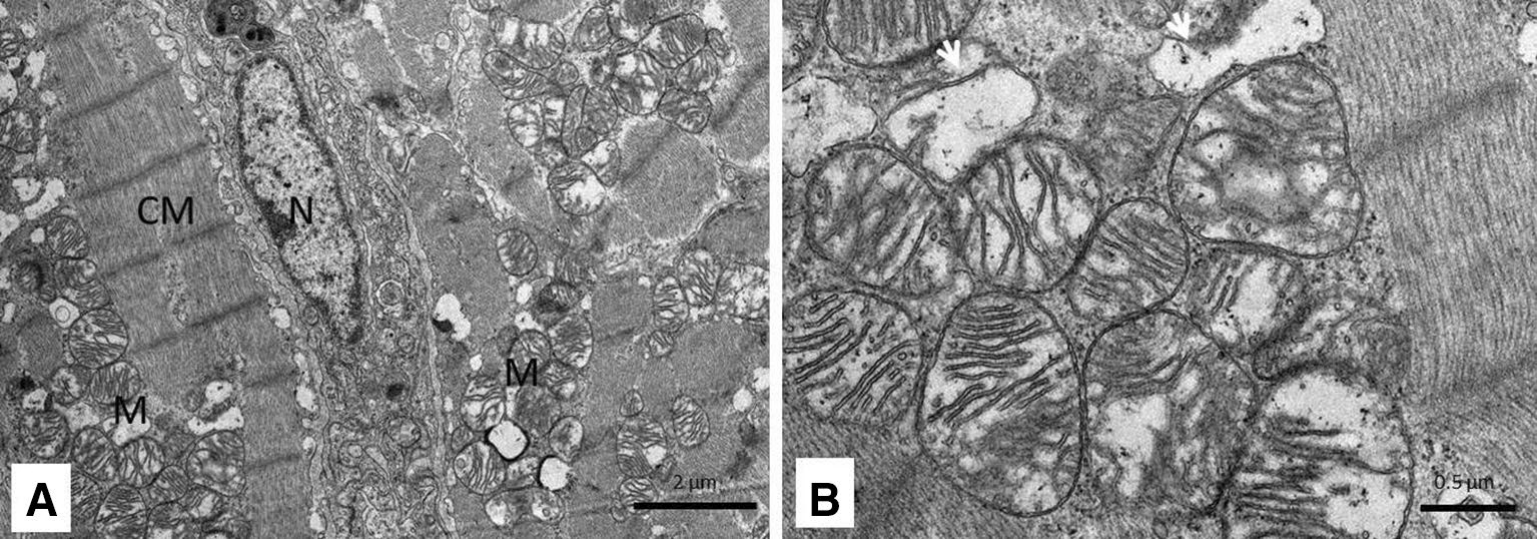
**Transmission electron micrograph showing mitochondrial damage in the myocardium of SAV infected Atlantic salmon myocardium at 11** **days post-infection.** **A** cardiomyocyte (CM) with enlarged mitochondria (M); nucleus (N) is still intact; **B** higher magnification of enlarged mitochondria with distorted cristae and completely devoid of cristae (white arrow).

**Figure 4:**
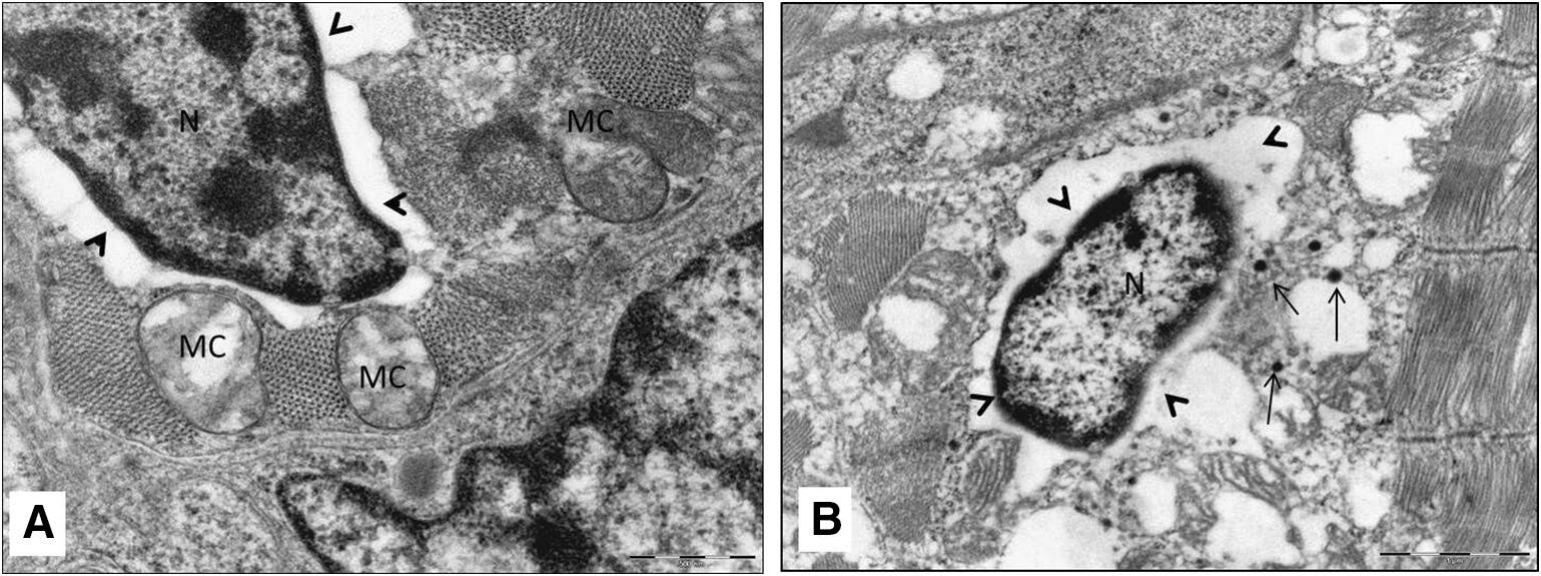
**Transmission electron micrograph showing nuclear damage in the cardiomyocytes of SAV infected Atlantic salmon at 11 days post-infection**. **A** High magnification of cardiomyocyte showing nucleus (N) perinuclear halo (arrow head) and damaged mitochondria (MC); **B** Damaged cardiomyocyte with intact nucleus (N) surrounded by enlarged perinuclear halo (arrow head), cytoplasmic vacuoles and electron dense virus like particles (arrow).

**Figure 5:**
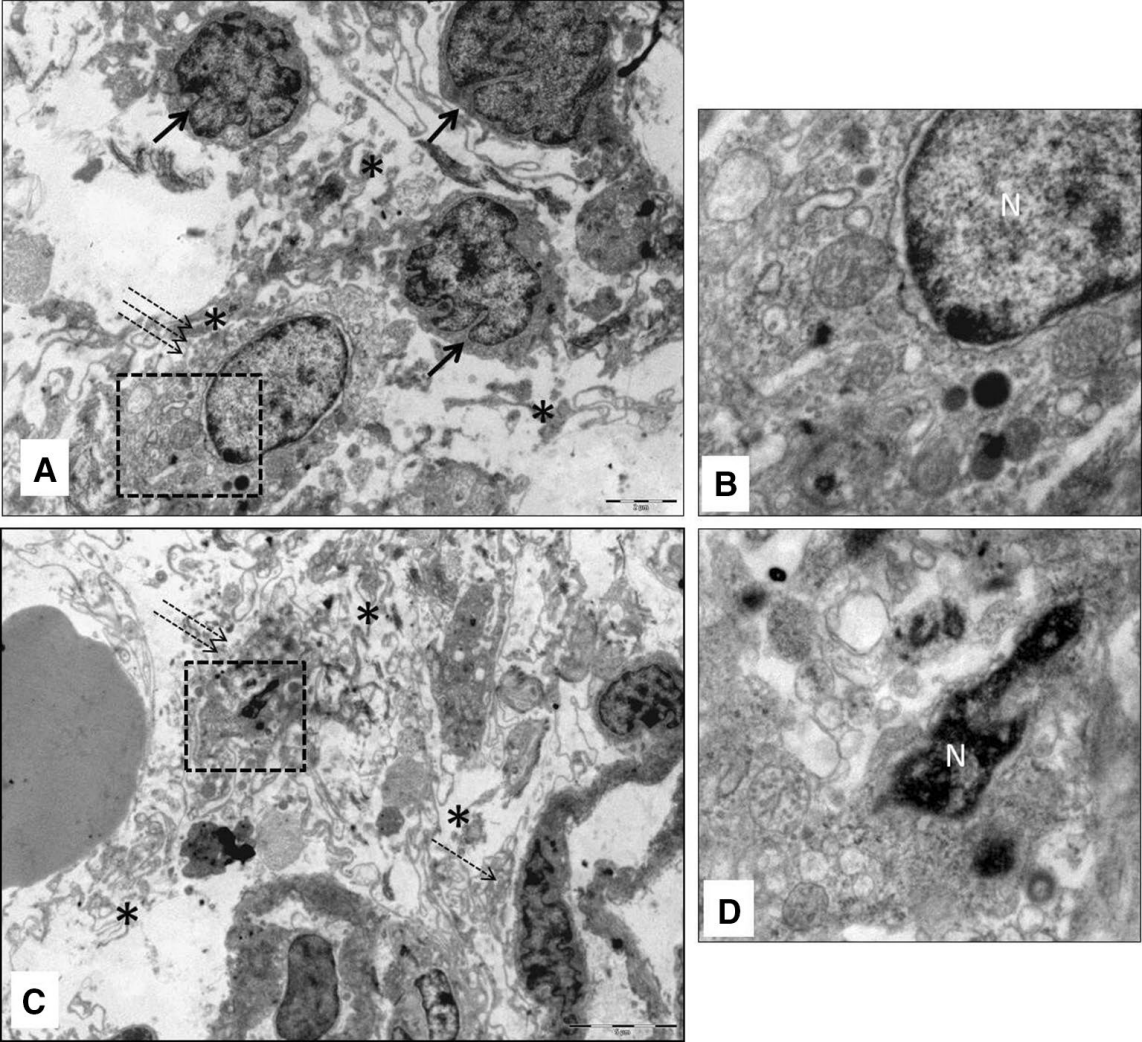
**Transmission electron micrograph of SAV infected Atlantic salmon myocardium 11 days post-infection**. **A** severely damaged cardiomyocyte (dashed arrows) with still-intact nucleus surrounded by markedly widened interstitial space (asterisk) and presence of leukocytes (arrow); **B** higher magnification of dashed square of (**A**) showing multiple membrane bound vesicles, possibly representing autophagy; **C** end stage cardiomyocyte with very scanty cytoplasm (double dashed arrows) and relatively intact cardiomyocyte (dashed arrow) with abundant interstitial space with cell debris (astrics); **D** higher magnification of dashed square of (**C**) showing markedly condensed chromatin in the cell nucleus that still has intact membrane. Note multiple cytoplasmic vesicles (autophagy vacuoles).

The TEM micrographs of the hearts of infected fish showed damaged myocardial cells. These cells lost features such as striations and intercalated discs (Figure 2A, B, C). In many cells microtubules were partially damaged, leaving parts of sarcomeres (Figure 2A). In some cells myo-filaments were completely dissociated, making cytoplasmic cross-striations indistinguishable (Figure 2C). Furthermore, cytoplasmic vacuoles of various shapes and sizes from very small to large were also seen in the damaged cells (Figure 2A–C). Cells containing cytoplasmic vacuoles became more abundant in 11 dpi infection compared to 4 dpi. The mitochondria in the damaged cells were enlarged and cristae were either distorted (Figure 3A, B), or in some cases, were completely absent (Figure 3B). Separation of the nucleus from the cytoplasm, leaving a perinuclear halo, was observed in TEM, especially at 11 dpi (Figure 4A, B). Electron dense particles that resembled alphavirus in size and shape were also noted in the vicinity of some of the damaged cardiomyocytes (Figure 4B). Mononuclear cells were seen in the heart, especially in areas of the myocardial damage (Figure 5A). On 11 dpi, some fish had severe myocardial cell damage characterised by loss of plasma membrane and cytoplasm, and also pyknosis (Figure 5C, D). The interstitial space of the myocardium also increased in area in regions where myocardial damage was severe and extensive.

**Figure 6:**
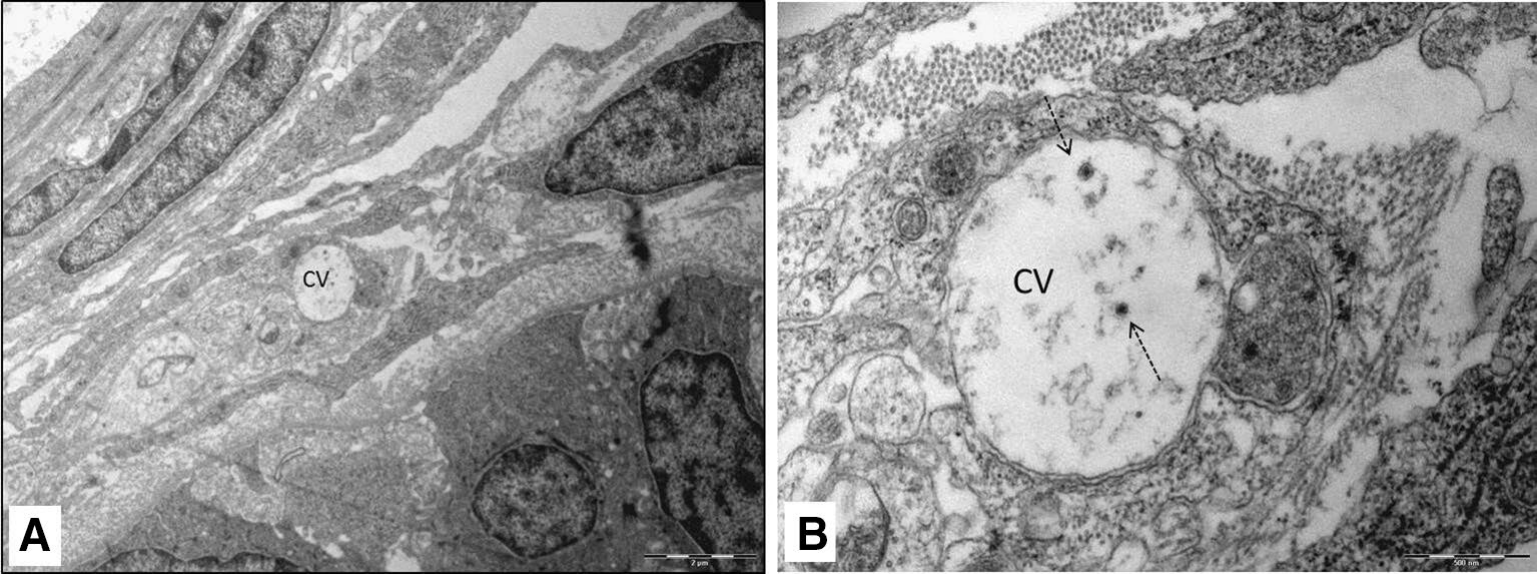
**Transmission electron micrograph of SAV infected Atlantic salmon gill epithelium 4 days post-infection**. **A** branchial epithelial cells with multiple vacuoles and, within one, double membrane vesicles (CV); **B** Cytopathic vacuole containing particles resembles ‘spherules’ with electron-dense centres (dashed arrow), representing possible type I replication complexes.

**Figure 7:**
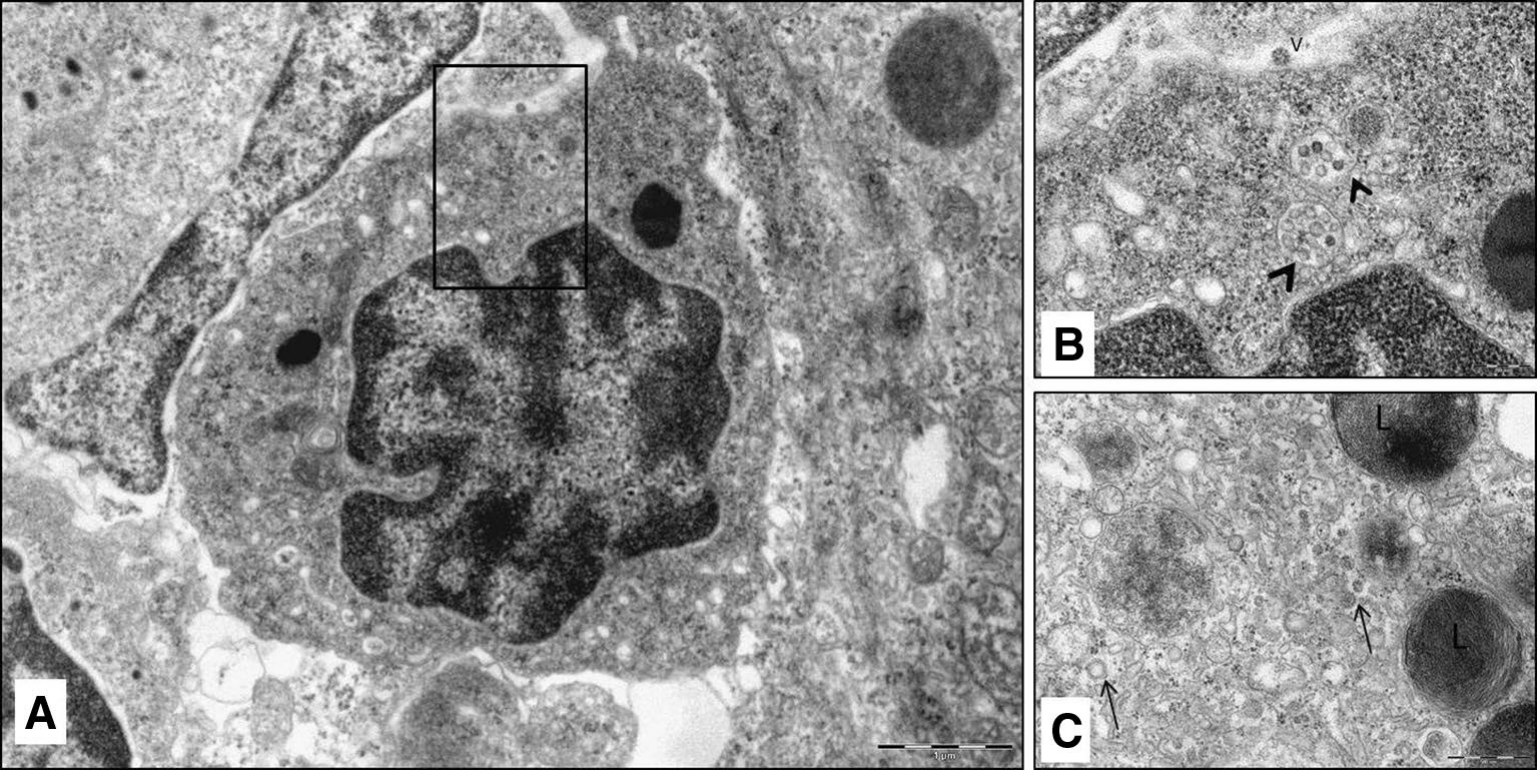
**Transmission electron micrograph of SAV infected Atlantic salmon head kidney 11 days post-infection**. **A** leukocyte containing numerous membrane-bound vesicles (bloked square); **B** endosome containing virus-like particles (arrow head) and free virion (v) near plasma membrane; **C** cytoplasm of leukocyte with small vesicular structures, bounded by electron dense particles at periphery, possible type II cytopathic vacuoles (arrow). Note lamellar bodies (L) in the vicinity.

In the gill, the cytoplasmic vacuoles observed under TEM were found mainly in branchial epithelial cells, however no cytolysis was observed (Figure 6). In the kidney, mild focal cytolysis was noted. Interestingly, membrane-bound electron-dense virus-like particles in the cytoplasm of some leukocytes in the interstitial cells in the head kidney were evident (Figure 7A, B, C). These membrane-bound vacuoles appeared to be early endosomes with phagocytosed virus particles.

## Discussion

Salmonid alphavirus is a newly emerged virus in farmed salmon that causes cardiomyopathy [11]. Previous studies have demonstrated that SAV can be detected in a wide range of host tissues, some of which have obvious histopathological lesions (target tissue–heart, pancreas, skeletal muscle), although the virus appears to persists for a long period of time in other tissues (e.g. gill) [2, 10, 12] without any apparent histopathological lesions. The information available regarding tissue tropism of SAV results from virology, molecular viral assays and histopathological studies. The degree of tissue involvement in virus replication, or mechanisms that govern such tissue tropism are not as well defined for SAV, however. The current study used a combined approach, employing virology, histopathology and electron microscopy to gain a better insight into tissue tropism and pathophysiology of SAV infection in Atlantic salmon parr under experimental conditions.

Reliably reproducing synchronised co-habitation challenges is difficult with SAV, and although this mimics the natural route of SAV transmission, it was not attempted in the present study. The i.p. challenge route used as an alternative method of viral transmission produced a successful infection in fish documented from the virology and molecular studies performed. This also allowed standardise the infective dose and thereby reduced variation between individual fish with respect to the more variable dose experienced in a co-habitation challenge.

The cell culture assay, based on virus isolation, conducted to establish presence of the virus and the RT-qPCR assays performed to quantify viral nucleic acid in samples, both detected SAV in heart, gill, and head kidney samples of infected fish. The number of fish found to be positive for virus was higher using the real time PCR approach compared to using conventional virus quantification by cell culture as expected. Although only a few heart homogenates produced a positive CPE in CHSE-214 cells, viral RNA was detected from all heart samples of virus challenged fish. Heart is generally considered to be as one of the best tissue for detecting SAV RNA and for virus isolation [12]. The low number of CPE positive heart samples in i.p. challenged fish was unexpected, possibly due to low levels of active, viable virus replication in the heart or could be as a consequence of the limitations encountered during sample processing. The tissues homogenates prepared from heart samples were five times more diluted than those from gill and head kidney (dilution factor 1:10), which may have reduced the sensitivity of the virus isolation by cell culture. The increased virus replication over experimental challenge seen in the kidney was in agreement with previous studies [22, 23]. Interestingly, all gill tissue homogenates produced a positive CPE on CHSE-214 suggesting that gill may be a useful tissue, both for SAV isolation, and also for monitoring purposes during naturally-occurring outbreaks. The gill was found to harbour SAV for prolonged periods of time in fish experimental infected with the virus based on the results of a nucleic acid based qPCR assay [12, 24]. The amount of viral nucleic acid detected in a sample does not necessarily reflect the number of infectious particles presents [25], as suboptimal synchronisation of genome replication and envelope assembly can lead to differential levels of defective infective particles, a situation which has been previously described for SAV and other alphaviruses [26]. Therefore, in interpreting pathogenic or clinical studies, the results obtained from cell culture assays may provide more accurate and meaningful information over molecular studies.

In general, mammalian alphaviruses target cells that are epithelial or fibroblastic in origin as well as some immune cells [27]. In fish, SAV is known to be permissive for various cells, including epithelial and fibroblastic cells [28, 29]. Although these in vitro studies have provided insight into infection kinetics, it does not mimic the virus preference in vivo.

In humans, depending on the clinical presentation, alphaviruses are categorised as encephalitic or arthritogenic alphaviruses [30]. The encephalitic alphaviruses that cause neuronal damage are more widespread in the Americas, while the arthritogenic viruses that give rise to more long-lasting articular and muscular diseases are found in the tropics and subtropics [27, 30]. In comparison, in salmon, SAV causes severe degenerative damage to the striated muscular tissue including skeletal muscle, oesophageal muscle and also heart muscle, but lesions in nervous tissues are rather uncommon [2].

In the present study, TEM examination of the heart, kidney and gills of the infected fish showed cytoplasmic vacuoles, a characteristic feature of tissues infected with alphavirus [31]. The myocardial damage seen in some infected fish was severe, especially at day 11 post-infection. The ultrastructure changes, such as disaggregation and lysis of myofibrils and chaotic distribution of mitochondria in the cytoplasm, are characteristics of acute damage to heart cells [32]. Severe destruction of myofibrils during infections in the heart can lead to dilated cardiomyopathy (DCM), both in humans and animals [33–35]. Immunological injury due to inflammation, direct or antibody-mediated cytotoxic damage or ischaemia can contribute to myocardial injury [33]. Mild to moderate inflammatory infiltration into the heart as reported in the present study, is a characteristic feature of SAV infection. Rodger and Michelle [36] reported round, flabby ventricles in the marine farmed salmon that had hitherto experienced a PD infection. Although, their report did not clearly indicate that PD was the primary cause for misshapen hearts, this does suggest that ventricular dilation seen in salmon recovered from PD could be a consequence of chronic SAV infection, which could however, be successfully visualised using TEM, similar to DCM in humans which results from alphavirus infection [34].

Myocardial changes present in individuals with DCM are difficult to characterise under light microscopy. However, TEM has been used to successfully visualised changes in the cardiomyocyte population of patients with DCM [37]. Although this application is not entirely relevant in fish, TEM was used to characterise SAV-associated cardiac damage in Atlantic salmon parr, changes in heart ultrastructure were found that might eventually have led to DCM in the fish. Moreover, TEM may help to compare and understand the pathology associated with other viral cardiomyopathies in salmon including heart and skeletal muscle inflammation and cardiomyopathy syndrome.

Although all three tissues studied showed features of SAV replication, the heart was the only tissue that showed free virus-like particles. They were seen freely in the cardiac tissues near damaged cells, although no virus particles were found budding through the plasma membrane, as seen in a previous in vitro study performed by Herath et al. [17].

While SAV was isolated from both kidney and gills, absence of free virus-like particles in the gills and the kidney under TEM could just be due to the fact that the area examined under TEM was devoid of virus particles, or perhaps virus usually isolated from these tissues might not be derived from the tissue itself, but from the blood supplying them. Nevertheless, this hypothesis should be experimentally demonstrated.

In the present study, membrane-bound organelles with particles resembling virus were frequently seen within renal interstitial leukocytes, suggesting possible SAV replication. SAV replication has also been seen within macrophage-rich head kidney cells of Atlantic salmon in vitro (Herath, un-published data). Furthermore, compared to other cell lines of fibroblastic and epithelial origin [28, 29, 38], TO cells, a commonly used continuous cell line produced from head kidney tissue of un-vaccinated salmon and containing a population of leukocytes [39], is highly permissive for SAV. Collectively, these observations strongly suggest tropism of SAV towards leukocytes, similar to that reported for other alphaviruses [30]. In central nervous system infections caused by Chikungunya virus, leukocytes are seen to actively migrate across the blood brain barrier helping the virus to reach nervous tissue [27, 31]. In salmon, SAV may use a similar mechanism to disseminate virus rapidly into tissues and also to reach peripheral tissues such as gills.

Although all gill samples of SAV-challenged fish induced a CPE in CHSE-214 cells and were positive by RT-qPCR, no free virus particles were observed in the gill epithelium under TEM. However, a few cells did have double membrane cytoplasmic vacuoles enriched with structures resembling SAV replication complexes [17], suggesting that SAV does indeed replicate in the gill epithelium. This also advocates that SAV replicates slowly in the gill, similar to ISAV which showed a low replication rate in primary gill cultures [38], thereby sustaining infection for longer periods, and allowing virus to shed easily into the surrounding water.

In summary, this study describes the ultrastructure of degenerative changes caused by SAV in the heart. Although no cytolytic changes were observed in the gills and the head kidney, which were also examined, these tissues may serve to sustain the SAV infection over a longer period. The absence of free virus-like particles in the gills and the kidney, as observed by TEM, suggests the possibility that the virus usually isolated from these tissues might not be derived from the tissue itself but rather from the blood supplying them. In this respect, this study has also demonstrated the possible tropism of SAV towards leukocytes.
